# Exposure: A Guide to Sources of Infection

**DOI:** 10.3201/eid1305.070180

**Published:** 2007-05

**Authors:** Sandra K. Schumacher

**Affiliations:** * Centers for Disease Control and Prevention, Atlanta, Georgia, USA

Exposure: A Guide to Sources of Infection is a dense reference book suited for health professionals and public health officials working within the realm of infectious diseases. This book does not use the typical format of solely detailing microbes in succession. Instead, it takes the novel approach of organizing microbes by sources of exposure, as stated in the title. Sections include animals, the environment, foods, humans, travel, and nosocomial infections. A listing of microbes is provided in the last section, followed by an exposure checklist in the appendix.

By compiling >250 pages of references, the author has tried to give a detailed and current review of the literature. Although some readers may not find this level of detail useful, it is interesting and does propel the reader to think beyond the usual microbes and their common routes of exposure. The author is particularly sensitive to the international nature of infectious diseases and has worked hard to thoroughly discuss cases and outbreaks that have occurred throughout the world. This is particularly evident in the last section of the book, which employs the more familiar format of listing microbes alphabetically. The author cites the effects of many infectious agents by detailing where the microbe is usually found, its prevalence, virulence, and mode of spread.

As the author states, the scope of the book is not clinical but rather epidemiologic. Therefore, this publication does not specifically provide suggestions for treatment and management decisions. However, this book stresses the need to be more conscientious of the many modes of infections, which may prompt a diagnosis that otherwise may have been missed.

